# The Importance of Embodiment and Agency in Parents’ Positive Attitudes Towards Shared Reading with Their Children

**DOI:** 10.1007/s10643-022-01415-1

**Published:** 2022-11-19

**Authors:** Natalia Kucirkova, Vibeke Grøver

**Affiliations:** 1 University of Stavanger: Universitetet I Stavanger, Stavanger, Norway; 2 Universitetet i Oslo, Oslo, Norway

**Keywords:** Parental attitudes, Shared book reading, Early literacy, Norway, Parents, e-books

## Abstract

Parents’ attitudes are an important indicator of whether and how parents engage in shared book reading (SBR) at home. This study analysed Norwegian parents’ attitudes towards reading books with their children aged between 1–4.5 years. Thematic analysis of data from 24 interviews revealed two main themes in parents’ accounts: agency (the child’s independence, the adult’s control as well as their shared control during SBR) and embodiment (physical presence and intimate experience of a SBR session). Both themes correspond to parents’ preference for reading print rather than digital books with their children. Findings are discussed from the socio-material theoretical perspective, with attention to their practical and policy implications.

## Introduction

Few other parent–child activities have received as much research attention and policy support in early childhood studies as the activity of adult–child shared book reading. Shared book reading (SBR) is considered a literacy activity par excellence for children learning to speak and read (Sénéchal, 2017). Pooled results from correlational studies show a significant impact of SBR on children’s emergent literacy and reading achievement (Bus et al., 1995; Scarborough & Dobrich, 1994) and meta-analytical studies show that SBR has an effect, though small, on children’s language skills (Dowdall et al., 2020; Noble et al., 2019). While there is a body of evidence about SBR benefits, mostly in the literacy domains, for pre-school children, limited evidence concerns adults’ views on SBR in families living outside of English-speaking countries. Our study addresses this gap with a theoretically-driven exploration of Norwegian parents’ attitudes towards SBR.

### Study Purpose

Inspired by the empirical literature documenting the positive benefits and diverse factors influencing SBR in families, we aimed to understand the key factors in Norwegian parents’ attitudes towards SBR with their children. For our study purpose, we analyse the ways in which Norwegian parents view story-reading experiences with their children in light of diverse book characteristics, and we follow the socio-material theoretical framework in our data analysis and interpretation.

### Literature Review

Given the relatively low implementation costs and high gains for families, the benefits of SBR have been translated into several intervention programs that train parents in language-stimulating and reading-promoting reading techniques. Dialogic reading intervention studies have showed positive effects on children’s vocabulary learning (Hargrave & Sénéchal, 2000), especially for the youngest (2–3-year old) children (Mol et al., 2008). In addition to training programs, SBR interventions centre on book-gifting schemes or book giveaway programs, which provide families with access to books and information about the importance of reading from an early age. The three largest book-gifting programs internationally—Reach Out and Read, Imagination Library, and Bookstart—rely on two main mechanisms: the provision of carefully selected books and coaching through written guidance for parents. The delivery mechanisms of books to children vary; in Reach Out and Read, books are provided to parents at paediatric controls, in Imagination Library families receive books through a mailing service, and in the Bookstart programs, book packs are distributed to families by librarians, early childhood practitioners/teachers or healthcare and community centres. The interventions vary in how many books children receive (e.g,. in Imagination Library children get a book every month while in Bookstart they get a reading pack at child’s birth or given milestone). The geographical coverage of book gifting schemes varies too; for example Reach Out and Read is most popular in the United States of America (USA) while Bookstart has been adopted in 15 European countries, as well as Asia, Oceania and North, Central and South America and Oceania), and recently, Norway. The international literature on Bookstart is relevant for our study given that the participants in our project took part in the Norwegian version of the Bookstart programme.

A recent meta-analysis found significant effect of Reach Out and Read, Imagination Library and Bookstart on children’s home literacy environments (De Bondt et al., 2020). The factors that contributed to the positive effects most were family participation in the intervention, particularly if the participation was voluntary and if it involved several information and reading demonstration sessions. Furthermore, the length of the intervention can increase the positive effects, with children’s long-term participation in the Imagination Library program showing largest benefits (Tura et al., 2021). In addition to evaluation studies of national book gifting schemes, book giveaway programs have been evaluated on a smaller scale, targeting specific groups of families. For example, the Family Literacy Bags program, which targets parents in the western USA with free books and reading guidelines in English and Spanish, found positive effects on parents’ perceptions of reading to their children in both English and Spanish-speaking families (Dever & Burts, 2002). Ready, Set, Share A Book!, program which was developed for 12-to 36-month-olds in the USA with emphasis on supporting parents’ dialogic reading strategies, improved parents’ reading skills and attitudes towards reading with their infants (Sally et al., 2022).

Despite the popularity of SBR in early literacy research and interventions, SBR is not universally practised. Book-gifting programs attempt to address SBR practices through the provision of material resources (e.g., access to books, guidance and training on how to read with children). However, SBR research show that family reading practices also depend on parents’ attitudes (parents’ beliefs, views and perspectives) about the importance of reading in children’s lives. In our study, we were interested in parents’ attitudes towards SBR as a little explored factor in diverse families.

### Parents’ Attitudes Towards SBR

Attitudes are overtly expressed, deliberate evaluations of a given phenomenon (see Arendt et al., 2019). Parents have diverse attitudes towards SBR and their attitudes correspond to their reading styles, habits and strategies at home (Barnyak, 2011; Bojczyk et al., 2016). A substantive body of SBR research has been dedicated to understanding parents’ SBR attitudes, particularly in relation to three key factors (see Fletcher & Reese, 2005): 1, the role of culture and family values; 2, children’s characteristics and literacy behaviour and 3, book and text-related factors, such as the book content and format. All three factors are relevant for our analysis of Norwegian parents’ attitudes towards SBR.

#### Family Characteristics

Culture plays a role in what parents think about SBR and how they engage in SBR with their children. While in some cultures, for example in Hong Kong, SBR is considered a vital part of responsible parenting (Wing-Yin Chow & McBride-Chang, 2003), in other cultures it is not. Differences between families are detectable even within the same country. For example, Surinamese-Dutch, Turkish-Dutch and Dutch mothers were found to hold different attitudes and use different kinds of reading styles with their children (Bus et al., 2000), and there were also different reading styles among immigrant families (African American, Dominican mothers, Mexican and Chinese mothers) in the USA (Luo et al., 2014).

Cross-cultural differences in SBR attitudes correspond to different reading styles: African American mothers tend to ask fewer questions but engage in more spontaneous verbalisation than Caucasian mothers (Anderson-Yockel & Haynes, 1994), while Peruvian mothers tend to deviate from the book text and engage in more storytelling during SBR than American mothers (Melzi & Caspe, 2005). In addition to culture, parents’ education and income levels uniquely predict SBR frequency at home, with lower SES families reporting reading less often than families with higher socio-economic status (Karrass et al., 2003).

#### Child Characteristics

Children’s age and child behaviour predict whether and how much parents engage in SBR at home, with studies showing that parents of younger children and children with high temperament being less positively inclined towards SBR at home (Vernon-Feagans et al., 2008). British early years practitioners reported and were observed to read less with children under the age of three (Boardman, 2020), suggesting that the youngest children might receive less SBR experiences than older children. Parents’ beliefs about whether their child is ready to benefit from SBR are reflected in how early they start reading with their children. Although children under the age of two have typically shorter focused and joint attention spans, there are well-documented benefits of parents reading to infants. For example, infants who were read at 8 months had higher language scores at 12 and 16 months (Karrass & Braungart-Rieker, 2005). A good illustration of the bidirectionality of effects is Bojczyk et al., (2016) study that found that mothers’ beliefs of how “ready” their child is to learn and benefit from SBR was directly linked to the SBR quality, which in turn mediated children's expressive vocabulary gains. Parents choose intentionally the types of books they read with their children in different contexts and at different stages in life. For example, English and Spanish speaking parents of children aged 9–18 months living in USA reported that reading board-books works best for this age group (Brezel et al., 2021).

#### Book Characteristics

Even though children benefit from both digital and paper books (for instance in terms of word comprehension and phonological awareness, see Korat et al., 2013), several studies show that parents strongly favour reading paper books. Indeed, parents’ attitudes towards digital books are less positive than towards print books (Strouse & Ganea, 2017) and this difference is reflected in the extent to which they facilitate digital reading at home. In a nationally representative sample with British parents, 76% of parents expressed strong preference for print over digital books to read with their children (Kucirkova & Littleton, 2016), and in a national sample of US parents, 53% of parents preferred reading print books for social reasons such as calming and bonding, even though a fifth of the surveyed parents reported that their child used e-books daily (21%) or several times a week (28% of surveyed parents, Etta, 2019). Observational studies of children reading digital books with their mothers showed differences in mothers’ type of support with different reading formats, with mothers facilitating reading of print books more actively than digital books but appreciating children’s high interest and engagement with digital books (Eggleston et al., 2021).

Understanding the impact of various factors on parents’ attitudes requires a theoretical framework that takes into account both the socio-cultural factors of family and child characteristics, and the physical or material factors of books and available reading resources in families.

### Theoretical Framework

An important conceptual framework for SBR studies addressing children under the age of 3 has been the adult–child-book framework developed by Fletcher and Reese (2005), which captures the conceptual orientation of SBR intervention and experimental studies conducted in 1990s and early 2000s. Fletcher and Reese’s (2005) framework is less well-suited for more recent studies, particularly observational and interview studies that examined the role of modern childhoods, digital media and complex socio-cultural relationships influencing parents’ SBR attitudes. Building on earlier conceptualisations of SBR from Vygotskian (1978) socio-cultural and Bronfenbrenner’s (1979) socio-ecological perspectives, the latter type of studies follow Bronfenbrenner’s recent theorisations that include a consideration of historical time (chronosystems, see Bronfenbrenner, 1992), neo-Vygotskian socio-cultural theory that explores intra-thinking among adults and children in non-hierarchical positions (Mercer, 1994) and to a large extent, the posthumanist tradition (Murris, 2016). Collectively, these theoretical orientations decentre the role of humans in family interactions and highlight the complex relationships between human and non-human and material and immaterial factors in understanding contemporary early childhood literacies (Pacheco-Costa & Guzmán-Simón, 2021). Inspired by this literature, we adopted the socio-material theoretical framework that is a compromise position between traditional and post-modern theoretical perspectives on SBR.

#### Socio-materiality

The socio-material theory has been at the forefront of educational studies in the last decade, with researchers taking a synergistic approach to social and natural influences in children’s development and education (Fenwick et al., 2015). Rooted in concepts originally developed in cultural historical activity theory, actor-network, spatial and material theories, socio-materiality is a conceptual response and critique of linear, dichotomous and reductive approaches to children’s learning (Heydon et al., 2015; Rowsell & Pahl, 2015). Socio-material researchers highlight that learning happens through transactions with material and human artefacts that are distributed across local and global spaces and that in their socio-material assemblages, provide learning opportunities and identity options (Kervin, 2016). The notion of entangled human-material interactions offers an understanding of literacy as a relational space that is open to children’s play, communication, belonging and language expansions in no pre-determined linear chain of events but rather grounded in relations between children’s interests and the opportunities of their environments.

In our study, we paid particular attention to the following components of the socio-material theory. First, we applied the socio-material lens to take into account multimodal, spatial and sensory materiality (see Mills, 2015) that might play a role in parents’ attitudes towards SBR. Second, aware of the role of social, or human, factors in SBR (the role of culture, family, child and parent characteristics) as well as the material characteristics (the role of books, the reading context and space) and their mutually constituting elements over historical and social time, we were keen not to impose any hierarchies on their value in our interpretation of parents’ attitudes and the reasons for these. From critical realism (see Mutch, 2013) and posthuman perspectives (Barad, 2007), socio-materiality has emerged as a useful tool to decentre the dominant role of humans and non-humans in interactions (Dunk, 2020; Fayard, 2017), with a direct attention to their joint influence in a unified “assemblage” (Johri, 2011). The third guiding component in our data analysis and interpretation was thus an understanding that socio-materiality does not privilege human or non-human factors but positions both as being part of the same larger ecosystem where digital and print reading inter- and intra-act, and where multimodal reading mediates the home literacy space.

### The Present Study

#### The Norwegian Context

Reading with young children is widely promoted in the Norwegian society, with dedicated Children’s Book Institute, Children’s Reading Charity and a range of government support schemes for libraries and reading in kindergartens and communities. Despite this policy support, evidence on Norwegian parents’ attitudes towards SBR is missing. The book-gifting intervention Bokstart was introduced as a pilot scheme in the Oslo municipality in 2020. The intervention was modelled after the British Bookstart model in terms of its focus on a free book pack to all families but the delivery of the books to families happened at healthcare stations, which is more similar to the Reach Out and Read model. Our study draws on interview data collected as part of the Bokstart’s evaluation. In conceptualising the study, we followed the theoretical framework of socio-materiality, which highlights the socio-material reasons in parents’ attitudes.

#### Study Aims

We were interested in identifying the key socio-material factors in the accounts of Norwegian parents, who participated in a Bookstart intervention, about reading with their children at home. Our analysis was guided by the research questions: What are Norwegian parents’ attitudes towards SBR at home? Which socio-material factors play a role in Norwegian parents’ attitudes towards SBR with their children at home?

### Methods

#### Study Procedure

The interview data was obtained through our research team’s participation in an evaluation of the Bokstart intervention in Norway. As part of the intervention, we conducted one-to-one telephone interviews with some of the parents participating in the Bokstart programs. The recruitment of these parents was facilitated through the health stations that were visited by the parents who received the Bokstart book package. There were no specific recruitment criteria other that the parents needed to have participated in the Bokstart intervention and had sufficient language skills to be interviewed in Norwegian. The health stations in the Oslo municipality distributed information about the study and consent forms to 27 parents. Our research team contacted all parents who consented to participate in the Bokstart evaluation and 24 agreed to be interviewed. The interview was conducted by phone by a research assistant who followed the same interview protocol for all parents.

#### Ethical Considerations

The study was assessed and approved by the Norwegian Centre for Research Data. The approved protocol followed strict data anonymisation and confidentiality processes, whereby the parents had the option to withdraw any quote attributed to them or their participation in the study at any time by informing the researchers. The participants’ consent was monitored on an ongoing basis during the telephone interview, which finished with a request for a quote that the participants were happy to share with the evaluation partner. This quote was then used in the Bokstart evaluation report. Our analysis drew on data from the full interviews. The interviews were professionally transcribed in Norwegian and quotes used in this paper were translated to English by the authors.

#### Participants’ Characteristics

Participants’ characteristics, including their children’s age, the language they speak at home and an approximate number of books they have at home, are in Table 1.

**Table 1:** Descriptive information of parents who were interviewed for the study

Nr.	The child’s age	Language of reading	Number of books at home
1	1,0	Norwegian and another language spoken at home	20–30
2	1,5	Norwegian and another language spoken at home	40–50
3	1,5	Norwegian	70
4	1,0	Norwegian	30–40
5	1,5	Norwegian	20–30
6	1,5	Norwegian	20–25
7	1,5	Norwegian and another language spoken at home	10–20
8	1,5	Norwegian and another language spoken at home	More than 100
9	1,5	Norwegian and another language spoken at home	5
10	1,5	Norwegian and another language spoken at home	50 +
11	2,5	Norwegian	30
12	2,5	Norwegian	50–100
13	2,5	Norwegian	20
14	2,5	Norwegian and another langauge spoken at home	Very many
15	2,5	Norwegian	40
16	2,0	Norwegian	30
17	2,5	Norwegian	6
18	2,0	Norwegian	25–30
19	3,0	Norwegian	40
20	3,0 and 1,0 (two children)	Norwegian and another language spoken at home	20
21	1,0	Norwegian	30
22	3,0	Norwegian	20
23	2,5	Norwegian	30
24	4,5	Norwegian and another language spoken at home	6–7

As can be seen in Table 1, all interviewees reported having some books at home, ranging from a minimum of five to more than a hundred books. Eleven children were aged under 2 years, nine under the age of three, and four under the age of five. The reading language was Norwegian for all families, but nine families also read in another language than Norwegian.

During the interviews, several of the parents mentioned that they had a profession that required frequent reading and a high level of competence (one parent specified that he worked as a teacher, and one as a project manager). Only one of the parents mentioned that they read both digital and printed books to the children; the rest of the interviewees talked about the reasons for why they read print and not digital books when they read with their child.

#### Thematic Analysis

Our analysis was theoretically guided by socio-materiality and methodologically by traditional thematic analysis. We used socio-materiality as a meta-perspective and in finalising the themes and their presentation in this paper, we paid particular attention to the consistency between the theoretical framework of socio-materiality and the presented analysis—a quality assurance issue highlighted in the original description of thematic analysis by Braun and Clarke (2006). In order to identify the key themes in parents’ interviews, we followed the classic procedure of a thematic analysis (Braun & Clarke, 2006; Clarke et al., 2015; Terry et al., 2017). The analysis was a flexible process with multiple reading and re-reading of the data in both Norwegian and English (both authors are fluent or native speakers of both languages), and deriving subjective interpretation of repeated immersion in data. The analysis findings are thus informed as much by the data as by our theoretical and disciplinary orientation to the data. In the Findings section, we present the outcomes of the thematic analysis and in the Discussion section, we comment on the outcomes from a conceptual and theoretical perspective.

### Findings

Perhaps not surprisingly (given the highly regarded status of SBR in the Norwegian society), all interviewed parents uniformly described SBR as an important activity for the child that they practice at home. As for the reasons for why they think so and what influences their SBR practice at home, we present two main themes informed by the socio-material theory. The theme “Agency” consists of sub-themes Child’s Control, Parent’s Control, Shared Control and The Technology’s Control. The theme “Embodiment” encompasses the sub-themes of Shared Presence, Intimacy and Physicality of Reading and Bonding. We use illustrative quotes for each theme, followed by the participant number (1–24).

#### Agency

Several parents mentioned the importance of their children being actively engaged and interested in the reading activity as an important SBR factor. The child’s agency, or control, during the activity was considered to be an important criterion for the parents’ positive attitude towards SBR. The parents referred to control when discussing whether the child enjoyed the session and learnt something from the reading activity. When describing a pleasurable SBR session at home, one mother (Participant nr.9) reported: *‘He [her 1.5-year-old son] is probably more there that he likes to just hold them [the books] himself and browse through them. And if mom does it with him, then he will have some quiet time in a way. Because the books are very large right. And because he has, the interest, in that they are big and colourful, so he would like to do it a little himself as well.’* Another mother of a two-and-half-year old son shared that when they read a book, the boy was actively contributing before and during the SBR session: ‘*Then he sits on my lap and then he brings a book… And he likes to flip in the books too. And he then flips through the book completely incoherently, that is to say… but since there are not much plot-based stories, to a large extent, it has not played that much of a role. But he is active, it is how he is like… how is like when he reads, he is active, yes’*. (Participant nr. 12).

Participant nr. 13, with a daughter of the same age as participant 12, explained that this active involvement is how learning from books happens: *‘I feel it helps her on her way to being able to express herself better and, because many of the books are a bit about her everyday things it might help her put a little more words into the things that happen in everyday life’.* In contrast to print books, the child’s control with digital books was not positively perceived by the parents, who felt that they should assume control of all digital media at home and keep children away from tablets and smartphones. The everyday struggles around this control negotiations were vividly described by participant nr.9: ‘*Yes then, we did read digital books, but it was such a struggle. He [the mother’s son] was more concerned with getting the tablet wasn’t he? You know when I read on my mobile or iPad, he is very curious about it and wants it himself then (…) Yes, so that's what's so dangerous about technology, that children want to control and own them. I'm trying to keep him away from technology.’*

#### Embodiment

A dominant theme in the interviews was the parents’ reference to the quiet and calm aspect of SBR at home. This framing corresponded with SBR’s description as a bedtime routine with exclusively print books. Reflecting on when and how they read, participant nr.10 described their SBR routine as an intimate moment of one-to-one conversation around the book when she switches off and the child enjoys her undivided attention: ‘*Yes, as I said, it is a break from everyday. We turn off the TV and turn off everything, phone, and I am a present with the kid. That's what reading is really for. Yes, I kind of feel like if I take a book and sit with her, then she likes it, and we are bonding. You know the child feels you are present, that's really what it is about.’*

The shared presence corresponds to shared attention to the story and the physical closeness that creates the feeling of intimacy and opens up space for bonding. As participant nr.12 put it: ‘*It's the physical closeness you know, that when you read to children, the children often sit on your lap or right next to you or something like that, so… And our attention is somehow so focused on the same thing, and that's it, really.’* The one parent in the sample (participant nr. 16) who reported reading both print and digital books with her daughter, commented on the calming effect of the particular digital book she had selected for her daughter: ‘*Yes, on tablets yes. It [the digital book] is called My Little Pony (…) it is with lyrics and music. It works very well. She listens and sits through the whole book.’*

Connecting to the notion of SBR as an intimate moment of shared presence, parent nr. 23 explained her preference for print books by stating: ‘*We did [read digital books] a little bit in the beginning before we went to buy books. Because it was so easy to just search online in a way… Then it became more that I just searched for stories and other stuff. But then I felt that it was not quite the same to sit and read on the phone or an iPad. It's kind of a little cosy to pull out a book and put away the phone in a way. There is a lot of phone use in a day. So it's kind of nice to put away the screen. I feel you get a little more contact and have a nicer time together then.’* The close connection moment in SBR was further underscored with parents making a contrast between the use of analogue and digital resources at home. Participant nr.12 revealed: *‘I think it's very like that, I think that when you kind of sit together and are directed towards the book together, it's very strong, there's a lot of connection in it. More than when we sit and watch, for example, something on TV. That it feels closer and more intimate and more like activating in a way I think.’*

## Discussion

Despite the strong body of evidence concerning the impact of SBR on children’s learning and its perceived value in the Norwegian society, there has been little research on Norwegian parents’ attitudes towards SBR at home. Previous empirical studies found that when parents read with young children it has a positive impact on their relationship with the child and motivates positive reading routines (Lee, 2010). Parents’ involvement in SBR positively impacts children’s reading acquisition (Sénéchal & Young, 2008) but this significantly depends on caregivers’ competence of reading books with their children (Dowdall et al., 2020). Parents from various cultural groups appreciate the opportunities to read with their children, as found, for example by Pandith et al. (2022) with parents from South Karnataka, India. Our study analysed parents’ attitudes from a socio-material theoretical perspective, with attention to the key factors that parents report when describing SBR at home.

Kucirkova (2021) reviewed studies concerning children’s reading of digital books with attention to the researchers’ epistemological perspectives on knowledge and learning and concluded that the empirical literature tended to separate material and social influences in children’s reading, with only theoretical studies acknowledging the socio-material entanglement. In this study, we adopted a socio-material lens to understand the extent to which parents report socio-material reasons for engaging in SBR with their children. We found that the interviewed parents had clear preference for print books when reading with their children and this preference was substantiated with their perception of SBR as an intimate and embodied activity. The parents wanted the child to be in control during the activity and described the child’s and their own agency during reading as instrumental in the learning process. We reflect on the study findings with reference to literature discussing the relationship between agency, embodiment and children’s reading practices.

### Child’s Agency with Digital and Paper Books

Children’s sense of control has been discussed in early childhood studies in relation to children’s rights (e.g., Berthelsen & Brownlee, 2005) and children’s volitional choice to participate in research studies, particularly from an ethnographic research perspective (e.g., Huf, 2013). Unlike in early childhood settings where children need to negotiate their agency with other peers and adults expected to teach and care for them (e.g., Sairanen et al., 2022), children’s agency at home is subject to negotiation with their family members. From the interviews it seems that during a SBR activity with print books, parents were willing to relinquish the control to the child but not so with a digital book. A similar tension was noted by Kucirkova and Flewitt (2022) with British parents, who, on one hand, were willing to support children’s agency and independent learning but on the other hand, wanted to be in control of how they engage with digital learning tools. Unlike in Kucirkova and Flewitt’s (2022) interviews, the Norwegian parents did not refer to children’s gradual introduction to digital books or possibilities for independent reading as they get older. While the interviewees recognised the unique assets of the digital books such as their low cost, interactive features, possibility of automatic narration and sound effects, they did not refer to digital books in terms of the often-cited creative, personalized, and authoring possibilities of digital books for young children (see Frederico, 2018; Undheim & Vangsnes, 2017).

Different formats are used for different reading purposes, with print books used more for shared reading and e-books for children’s independent reading (Etta, 2019) and they come with different affordances for reading, with e-books designed for learning or entertainment and print books also for literary and aesthetic experiences. The fact that parents rarely perceive these features of digital books as beneficial for their SBR practice corresponds to questionnaire and interview data from other studies; for example Australian mothers of two-year-old children who reported strong preference for print books, especially for bedtime routines (Nicholas & Paatsch, 2021).

#### Embodiment

At the beginning of the digitization turn of early literacies, Mangen (2010, p. 416) drew attention to the ‘potential impact of the intangibility of the digital’ and has since richly theorized and empirically documented the connections between reading fictional narratives and the affordances of the digital medium. Within the interdisciplinary paradigm of embodied cognition, reading is considered to involve the whole body and the difference between reading on paper and digital is explained by the differences in the physical interaction between the material properties of the books and the reader (Mangen & Van der Weel, 2016). This perspective corresponds to the Norwegian parents’ attitudes towards the affordances of the digital books for SBR with their youngest children. It also connects to literature that emphasises the pleasurable aspects of reading and the hedonic nature of bodily and physical engagement with texts and the sense of place and presence they create. Mackey (2022) refers to the “private pleasures of reading” (p. 3) and details how embodied engagement with texts facilitates a sense of presence during reading. Extrapolating these theoretical perspectives onto the parents’ accounts, we conclude that the connection between body, a hedonic perception of reading and the sense of presence, remains an important, and thus far, overlooked, dimension of parent–child SBR.

### Study Limitations

Our sample of participants was small and drawn from a particular group of Norwegian parents, namely those who participated in the Bokstart intervention, which targeted parents of infants with a pack of two free print books and dedicated guidance on the importance of SBR for families. Parents’ participation in the Bokstart programme may have affected their overall attitudes towards SBR. Moreover, the participant interviews were conducted close to the Covid-19 pandemic and parents’ experience of SBR may have been clouded by the recent lockdown experience and potentially, the increased media usage during that period. The findings need to be interpreted as qualitative interview data and future research needs to expand our findings in relation to diverse families and measures of the relationship between parents’ attitudes and SBR interaction quality on children’s outcomes. In particular, parents’ attitudes towards SBR could be explored in relation to class, linguistic or ethnic diversity in families.

### Study Implications

Given the documented relationship between parents’ positive attitudes towards SBR and observations of actual practice (Barnyak, 2011), our findings carry some important practical and policy implications. The parents’ emphasis on the child’s and their own control during SBR speaks to intervention studies concerned with parents’ confidence and guidance during reading, particularly with atypical or struggling readers (e.g., Bailey et al., 2022). Responsive and shared control between parents and children during SBR was shown to support higher learning benefits for pre-school children (Landry et al., 2012). Furthermore, programs that capitalise on children’s agency document children’s positive attitude towards SBR and dovetail with parents’ attribution of child’s control to the success of a SBR session. As a way of an example, in an intervention with 142 families of 3–6-year-olds in Czechia, children’s agency, particularly children’s volition during SBR, was the highest rated by the parents and more important than the number of children’s books at home or parents’ SES characteristics (Gavora, 2021). We highlight the importance of control in relation to the embodiment theme that came to fore in parents’ narratives. Physical interaction around the book affords an opportunity for bonding, sense of shared presence, and a quiet and pleasurable moment, which are attributes in short supply in modern families. Our findings indicate that Norwegian parents value SBR as an activity conducive to such moments and we encourage future SBR programs to explore this dimension in more detail. It would be particularly interesting to examine how book gifting programs, which thus far, have emphasised the language-related benefits of SBR, could respond to this hidden value of SBR.
